# The effects of gender, educational level, and personality on online learning outcomes during the COVID-19 pandemic

**DOI:** 10.1186/s41239-021-00252-3

**Published:** 2021-04-02

**Authors:** Zhonggen Yu

**Affiliations:** Department of English Studies, Faculty of Foreign Languages, Beijing Language and Culture University, 15 Xueyuan Road, Haidian District, Beijing, 100083 China

**Keywords:** COVID-19 pandemic, Educational levels, Gender, Personality

## Abstract

With the rampant pandemic of COVID-19, an increasing number of people are acquiring knowledge through online learning approaches. This study aims to investigate how to improve online learning effectiveness during this special time. Through a mixed design, this study revealed the effect of educational levels, gender, and personality traits on online learning outcomes. It was concluded that postgraduates (N = 599) outperformed undergraduates (N = 553) in online learning, learners (N = 1152) with strong personality traits such as agreeableness, conscientiousness, and openness to a new experience outperformed those with strong extraversion and neuroticism. Future research could improve interpersonal interactions and encourage learners to post words in the online discussion forum, focus on how to design scaffolding online learning and how to improve the quality and dynamic of the online contents, and highlight blended learning rather than either merely online or traditional face-to-face learning.

## Introduction

With the rampant pandemic of COVID-19, an increasing number of people are acquiring knowledge through online learning methods. The purpose of the study is to identify the influence of personalities and demographic variables on online learning outcomes in the COVID-19 pandemic.

### Benefits of online learning

The recent decade has been witnessing dramatic growth and various benefits in the use of online learning in education (Allen & Seaman, 2017). A great number of students are taking their courses online, which urged teachers to design online courses to improve learning and teaching effectiveness (Evans, 2014). Numerous studies reported that online learning could increase student participation, improve discussion quality, and foster online interactions. The discussion forum could support students and improve learning by solving difficult problems. Mobile technologies such as applications and computers could enable an easy access to an online learning platform and facilitate mobile learning effectiveness (Panigrahi et al., 2018).

Collaboration and virtual community could be established in the online learning context. Online learning, assisted with information technologies such as laptops, tablets, iPads, and mobile phones, has been widely used and well accepted in higher educational institutes (Starr-Glass, 2013). Online learning could bring numerous benefits to learners, e.g. diverting students’ attention to important knowledge and enabling them to engage in collaborative learning activities (Alwi et al., 2012). Collaborative learning was strongly and positively correlated with peer discussions and engagement rates (Brown, 2001). Formation of virtual communities could benefit online learning outcomes (Panigrahi et al., 2018).

### Challenges of online learning

Disadvantages of online learning exist in learner engagement, academic success, and time-consumption, despite that numerous studies have reported the effectiveness of online learning compared with traditional learning (e.g. Bernard et al., 2004; Means et al., 2013a, 2013b). A large number of teachers still resisted the use of an online pedagogical approach and believed that online learning could decrease the engagement of students, thus leading to less favorable academic success than traditional face-to-face instruction (Lederman, 2018). Very few online teaching and learning activities could follow a rigid schedule and design (Tallent-Runnels et al., 2006). Through the traditional approach, excellent teachers could attract students and hold their interest more than through the online approach (Garson, 1998). Online teaching could be more time-consuming than traditional teaching (Cavanaugh, 2005).

### The necessity to conduct this study

Given both benefits and challenges, it is necessary to study the effectiveness of online learning which is especially widely used during this difficult COVID-19 pandemic time. Scanty studies have focused on the effects of demographic variables and personalities of learners on online learning. This study will thus examine their effects in the online context and provide constructive suggestions to improve the online learning effectiveness.

## Theoretical framework

There are numerous acceptance models of personality traits, among which a widely accepted model is the five-factor model (FFM) (Costa & McCrae, 1992). FFM divided personality traits into five dimensions, i.e. extraversion, agreeableness, conscientiousness, neuroticism, and intellect/imagination experience/openness to a new experience.

The Big Five Model (BFM) was used to identify the correlations between the personality of learners and their perceptions of the online learning method (Arispe & Blake, 2012). BFM, a well-accepted psychological model, was a taxonomy classifying personality traits into agreeableness, conscientiousness, extraversion, neuroticism, and openness to a new experience (John et al., 2008). Agreeableness refers to concepts such as trusting, politeness, tolerance, and willingness to cooperate. Extraversion indicates the degree of individual sociability and assertiveness. Conscientiousness indicates the degree of individual responsibility, reliability, endurance, and perseverance. Neuroticism indicates the degree of individual anxiety, depression, and insecurity. Openness to a new experience indicates the degree of individual curiosity, creativity, and open-mindedness (Barrick & Mount, 1991). This study will use BFM as a theoretical framework to explore the influencing factors in online learning.

## Literature review

Since the outbreak of COVID-19, there have been many studies committed to online learning. Most of the studies have reported positive online learning effectiveness during the COVID-19 pandemic. The online, indoor, and desk-based learning could benefit secondary students and enable them to learn effectively and continually during the COVID-19 pandemic lockdown (Van Haeften et al., 2020). Online learning via the Community of Inquiry framework (CoI) could greatly increase students’ engagement in learning and improve learning achievement and team instruction during the COVID-19 pandemic (Tan et al., 2020). Medical student were ready to learn through the online and synchronized model, indicating the future model of medical education, whose effectiveness might be ensured based on a rigorous framework (Khalil et al., 2020). Online learning enabled Ophthalmology students to learn at any place, at any time and on any device although it still had numerous challenges (Kaup et al., 2020).

Researchers have proposed constructive suggestions for online learning improvements. For example, suggestions were proposed to enhance online learning of undergraduate students during COVID-19 by establishing an upper-level, project-based biochemistry laboratory class (Zewail-Foote, 2020). Through Facebook group Strategies for Teaching Chemistry Online, suggestions were raised regarding how to learn and teach through online learning based on the Technological Pedagogical Content Knowledge framework (DeKorver et al., 2020). Online learning advantages included remote learning, comfort, and accessibility but online learning was limited to inefficiency and difficulty in supervising students (Mukhtar et al., 2020). When conducting online teaching, teachers could try to monitor students and improve their learning efficiency.

Online teachers could also notice various influencing factors in online learning. In the online learning during the pandemic, undergraduates’ anxiety was negatively related to foreign language enjoyment. Their coping behaviors, negative, and positive emotions were closely related and coexisted in online learning during the pandemic. Online resources, as well as retrospective and general enjoyment, greatly influenced their coping behaviors and stressors (Maican & Cocorada, 2021). It is, therefore, important for online course designers to pay enough attention to these interweaving factors.

However, online learning could bring about negative results regarding health and students’ attitudes. Children could catch eye strain due to frequent engagement in online learning in the COVID-19 time (Mohan et al., 2021). Algerian university students, who preferred the traditional pedagogy, negatively evaluated online learning and felt reluctant to accept the online model during the COVID-19 pandemic (Blizak et al., 2020).

Nevertheless, very few of previous studies have examined the effect of gender, the educational level, and personalities on online learning effectiveness, let alone in a Chinese context. This study, centering on the effect of the gender, the educational level and personalities on online learning outcomes in the Chinese context, is thus considered meaningful and important.

### The gender and educational level

The COVID-19 pandemic has been witnessing a growing number of online learners with heterogeneous demographic backgrounds in terms of gender and educational levels.

Several studies have investigated the effect of learners’ demographic backgrounds on online learning outcomes (Gašević et al., 2016). Numerous studies have explored the impact of gender (e.g. Boyte-Eckis et al., 2018; Cai et al., 2017) and educational levels (e.g. Diep et al., 2016) on online learning outcomes. Educational levels could greatly predict online learning outcomes (Huang & Fang, 2013), while the effect of gender on online learning outcomes is controversial.

Females could achieve higher learning outcomes than males because they were more persistent and committed than males (Richardson & Woodley, 2003). Females had stronger self-regulation than males, which also led to their significantly more positive online learning outcomes than males (Alghamdi et al., 2020). However, no significant gender differences were revealed in leaning outcomes because males were more stable in attitudes, while females performed well in engagement (Nistor, 2013). Furthermore, no significant gender differences in learning outcomes were found based on learning styles. There were also no significant gender differences in the learning satisfaction of online millennial learners (Harvey et al., 2017). Given the inconsistent findings, we proposed the following alternative hypotheses:

**Hypothesis 1.** The gender of learners is significantly and strongly correlated with online learning outcomes.

**Hypothesis 2.** The educational level is significantly and strongly correlated with online learning outcomes.

### Definition of personalities of learners

An earlier definition of personality was provided by Funder (1997: 2) as an individual characteristic pattern of thought, emotion, and behavior, behind which existed psychological factors and connections. Personality has recently been defined by McGeown et al. (2014: 279) as several potential traits that influence individual behaviors, thoughts, and feelings. The latest definition of an individual’s personality has been the stable cluster of traits and styles that an individual possesses, including dispositions (i.e., natural trends or individual inclinations) and the style the individual differs from the community (Bergner, 2020). We comprehensively reviewed the literature and took into consideration five factors of personality traits, e.g. agreeableness, conscientiousness, extraversion, neuroticism, and openness to a new experience (John et al., 2008).

Recent years have been witnessing an increasing number of studies on learners’ personality traits in the context of online learning. Personality traits of learners were an important factor that could influence the effectiveness of online learning (Varela et al., 2012). Personality could greatly influence online learning success in terms of final grades and retention rates (Meredith, 2011), as well as the online and blended approach-based learners’ satisfaction (Bolliger & Erichsen, 2013). Personality could also significantly influence online learners’ attitudes towards the online learning approach rather than academic achievements (Kelly & Schorger, 2002). A further study (Keller & Karau, 2013), based on engagement, value to career, overall evaluation, anxiety/frustration, and preference for online courses, revealed significant correlations between learners’ personality and their perception of online learning. It is thus essential to identify personalities in the context of online learning.

### The role of personality

Personality plays an important role in the learning context. Learners with different personality traits prefer different educational approaches. Some might prefer a face-to-face traditional approach, while others might prefer an online learning approach or blended approach (Bolliger & Erichsen, 2013). Learners’ personalities could predict their satisfaction (Pawlowska et al., 2014), dropout rate (Alarcon & Edwards, 2013), learning motivation (Zhou, 2015), and academic success (Vedel, 2014). Personality traits potentially influenced collaborative learning effectiveness and quality (Kichuk & Wiesner, 1997).

Numerous studies have explored the effect of personality traits on academic performances (e.g., Kichuk, & Wiesner, 1997). Nevertheless, most of these studies recruited participants from the same venue and there was a lack of studies on the effect of personality traits in online learning contexts, especially in synchronous verbal communicative situations (Lara, 2013). Worse, no substantial empirical research could demonstrate whether extraversion, introversion, and anxiety could hinder or foster online learning effectiveness (Abe, 2020). Considering previous findings, we propose the alternative hypothesis as follows:

**Hypothesis 3. **The personality is significantly and strongly correlated with online learning outcomes.

### The role of extraversion

Moreover, specific personality such as extraversion greatly influences online learning outcomes. Extroverted or sociable learners outperformed those who were introverted or less sociable (Bell, 2007). Extraverts and ambiverts tended to feel uneasy due to the isolated context in online learning, and they thus preferred a face-to-face or a blended learning approach. By contrast, introverts, reflective, and thoughtful learners tended to like the asynchronous and self-regulated online learning approach since it did not require lots of group work or collaborative tasks (Fuster, 2017). Another supportive research was that introverts might prefer an asynchronous online learning approach where they could learn at their own pace (Bhagat et al., 2019).

**Hypothesis 4.** The level of extraversion is negatively correlated with online learning outcomes.

### The roles of neuroticism and conscientiousness

Conscientiousness was considered the most robust predictor of the personality of learners. Learners with different personality traits could hold different attitudes toward online learning. Learners with stronger conscientiousness and intellect or imagination could more likely positively evaluate online learning than those with less, whereas those with stronger neuroticism could more likely negatively evaluate online learning (Bhagat et al., 2019).

Specific personality traits exert a great influence on the correlations between perceived worthiness and the intention to engage in online learning. The personality traits such as neuroticism moderated the correlations between the perceived financial worthiness and the intention to join online learning. Neuroticism was considered the only trait influencing the effect of perceived emotional value on the intention to join online learning. Different degrees of personality traits exerted different influences on the effect of perceived value on the intention to participate in online learning (Watjatrakul, 2020). Learners with strong conscientiousness could arrange their learning activities in the course of semester, which improved their learning outcomes (Theobald et al., 2018). Therefore, we proposed the following two alternative hypotheses:

**Hypothesis 5.** The level of neuroticism is negatively correlated with online learning outcomes.

**Hypothesis 6.** The level of conscientiousness is positively correlated with online learning outcomes.

### The roles of agreeableness and openness to a new experience

An increasing number of studies have been committed to the correlation between learners’ personalities, their satisfaction, and learning outcomes in online learning contexts. Personality traits such as openness to a new experience and agreeableness could greatly influence the online learning outcomes. Learners with similar personality traits, e.g. openness to a new experience and agreeableness could prefer a similar online learning context where their learning outcomes could be improved (Cohen & Baruth, 2017).

Personality traits such as agreeableness and openness to a new experience could greatly influence the evaluation of the perception of a career (Bhagat et al., 2019). Learners with strong agreeableness tend to be optimistic and deem peers and teachers as cooperative friends (Karim et al., 2009). Learners with openness to a new experience often hold positive attitudes toward emerging online technology-assisted learning (Zhou & Lu, 2011). Thus, we proposed the following two alternative hypotheses:

**Hypothesis 7.** The level of agreeableness is positively correlated with online learning outcomes.

**Hypothesis 8.** The level of openness to a new experience is positively correlated with online learning outcomes.

## Research methods

This study adopted a mixed-design research method to analyze both quantitative and qualitative data obtained from two scales and a semi-structured interview respectively. The dependent variable is learning outcomes, and the independent ones are the general personalities, the levels of extraversion, neuroticism, openness to a new experience (or Intellect/imagination), agreeableness, and conscientiousness, as well as demographic variables such as gender and educational levels.

### Participants

We randomly recruited participants (N = 1152) from a public university in China, who, majoring in languages, received online education of various courses for a semester during the COVID-19 pandemic. Females (N = 595) slightly outnumbered males (N = 557) because language majors tend to be female-dominant. Participants involved both undergraduates (N = 553) and postgraduates (N = 599), ranging from 18 to 25 years old. The online courses they learned included *An Introduction to Linguistics, Intensive English Reading, Extensive English Reading, English Writing, English Speech and Debate, English Grammar, An Overview of English-speaking Countries, Advanced English Reading and Writing, A History of British and American Literature, English-Chinese Translation, English Interpretation, Western Literary Criticism, Literary Translation, Lexicology, Cross-cultural Communication, English History, An Introduction to Western Culture, Selected Readings of British and American Literature,* and *English News Listening and Speaking,* etc*.* All the participants have received online learning for at least 4 months continually and all of them were voluntary to participate in the research.

### Research instruments

*A Big Five Scale (BFS)* (McCrae & Costa, 1995). BFS aims to determine the levels of five factors, i.e. extraversion, neuroticism, openness to a new experience (or Intellect/imagination), agreeableness, and conscientiousness. Each factor was identified by eight to nine questions (see Appendix A), e.g. *I see myself as someone who prefers to be alone; I see myself as someone who is not easily bothered by things; I see myself as someone who does things I later regret.* Each question is followed by a five-point Likert Scale, ranging from *Disagree strongly* to *Agree strongly.*

Cronbach’s alpha coefficients were reported satisfactory by Donnellan et al. (2006) regarding the scales of extraversion (α = 0.82), agreeableness (α = 0.75), conscientiousness (α = 0.75), neuroticism (α = 0.70) and openness to a new experience (or intellect/imagination) (α = 0.70). All of the values reached a satisfactory level. Furthermore, Cooper et al. (2010) demonstrated that BFS was internally consistent and concurrently valid in the Mini-International Personality Item Pool and reported that Cronbach’s alpha coefficients of extraversion (α = 0.81), agreeableness (α = 0.70), conscientiousness (α = 0.68), neuroticism (α = 0.72), and intellect/imagination (α = 0.70) reached satisfactory levels. The Cronbach’s α values in this study also reached a satisfactory level of the scales of extraversion (α = 0.75), agreeableness (α = 0.76), conscientiousness (α = 0.80), neuroticism (α = 0.78), and openness to a new experience (α = 0.81).

*A scale to determine online learning outcomes*. Generally, learning outcomes are comprised of six dimensions, i.e. assignments, sign-in, audio and video watching progress, chapter learning times, discussions, and tests.

Specifically, assignments, accounting for 20%, indicate the average score of all tasks. Sign-in accounts for 5% of the total score, each sign-in obtaining 1 point. The number of 30 sign-in times reaches a full score. Audio and video watching progress accounts for 20%. The completion of the video/audio watching leads to full marks, and the score of a single video/audio will be equally distributed, with the full mark of 100. Chapter learning times, accounting for 10%, lead to a full mark when students learn chapters for over 300 times. The discussion accounts for 10%. Posting or replying to a discussion obtains 2 points, a *like* obtains 1 point, and the full mark is 100 points. Tests, accounting for 35%, are the average of all test marks.

*Interviews to collect qualitative data*. A semi-structured interview was designed to collect qualitative data. The interview consists of three sections. Section One aims to collect demographic data, such as ages, educational levels, and online learning experiences. Section Two is the body part including several questions to collect data regarding their personality traits and online learning outcomes. Examples are “What personalities do think you have?”, and “What do you think of your online learning outcomes?”. The last section aims to obtain their consent forms and extend gratitude to the interviewees. Due to the COVID-19 pandemic, we carried out the interviews through video conferences and blank fillings rather than face-to-face communication. In this way, more interviewees (N = 102) were recruited online than the face-to-face model. The research instruments are summarized in Table 1.

**Table 1:** Research instruments

Research instruments	Components				
BFS	Extraversion	Neuroticism	Openness to a new experience	Agreeableness	Conscientiousness
Learning outcomes	Assignments	Sign-in	Audio and video watching	Chapter learning times	Discussions and tests
Interviews	Video conferences; Blank filling				

### Research procedure

**Fig. 1:**
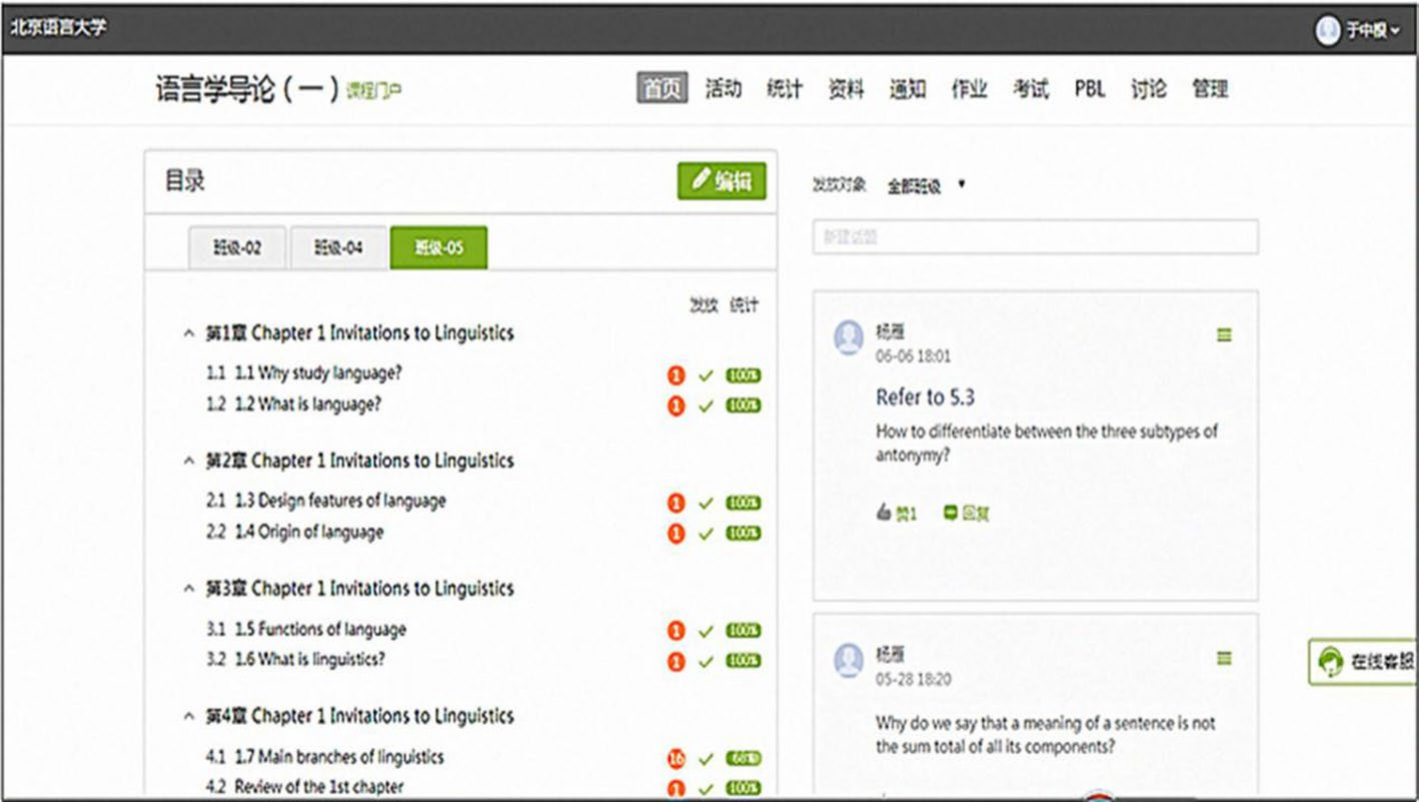
BLCU MOOCs of the course “An Introduction to Linguistics”

The online education was conducted via BLCU MOOCs and Superstar Learning System (Fig. 1). The former is an online learning platform designed by experts in the public university, while the latter is a mobile application developed by Superstar Company. On the computer, learners and teachers could access BLCU MOOCs, while Superstar Learning System was installed on a mobile device. Both systems are integrated into an entity where identical contents and similar functions are provided.

As shown in Fig. 1, teachers could complete various tasks to encourage students to engage in learning activities. They could design the learning contents such as Chapter 1 to Chapter 4, which can be accessed conveniently by clicking the target icon. The teachers could organize online learning activities such as registering, polling, question answers, discussions, quizzes, and grouping tasks, etc. They could summarize and analyze learning outcomes such as assignments, tasks, scores, activities, discussions, tests, engagements, and exams. They could upload rich resources to the platform in various forms, e.g., videos, audios, texts, and lecture notes. They could allot assignments to students and send alerts to those who failed to complete the assignment in time. They could manage students by adding or deleting the number of students. They could choose teaching assistants and organize teaching teams.

Students could improve their learning outcomes by actively participating in various learning activities. They could download a sea of learning resources from the database and Internet, complete assignments based on the requirements, watch videos, listen to audios, learn academic contents based on the lecture notes, review their performances, check their assignment marks, and take quizzes, mid-term or final exams. They could join learning activities, share opinions by group discussions, resort to peers or teachers for difficult problem solutions, and sign in or drop out of a course. They could start a topic for peer discussion. They could also check their assignment scores and review the learning progress.

**Fig. 2:**
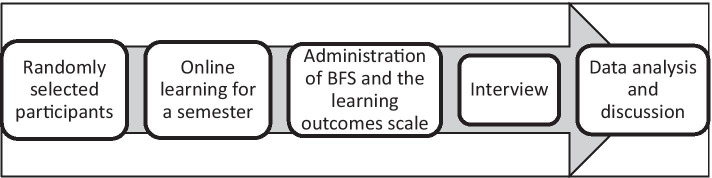
The research procedure

After students’ 4 months’ online learning, BFS and the learning outcomes scale were administered to them to identify the levels of extraversion, neuroticism, openness to a new experience, agreeableness, conscientiousness, and learning outcomes. Then we conducted the interview via video conferences and blank fillings. We recorded the video conferences, transcribed and then analyzed the data from both transcriptions and blank fillings (Fig. 2).

## Results

### The descriptive data analysis

The descriptive data analysis is shown in Table 2. The dependent variable is *learning outcomes,* while the independent variables include *gender, educational levels, extraversion, agreeableness, conscientiousness, neuroticism,* and *openness to a new experience.* The normal distribution was tested via the skewness and kurtosis, which met the assumptions of linear regression analysis since their values ranged from |3| to |10| (Kline, 2005).

**Table 2:** Descriptive data analysis

Variables	N	Minimum	Maximum	Mean	Std. deviation	Skewness		Kurtosis	
	Statistic	Statistic	Statistic	Statistic	Statistic	Statistic	Std. error	Statistic	Std. error
Gender	1152	1	2	1.52	.500	− .066	.072	− 1.999	.144
Edulevel	1152	1	2	1.52	.500	− .080	.072	− 1.997	.144
Extra	1152	25	39	28.97	1.789	1.755	.072	6.731	.144
Neuro	1152	20	33	26.24	1.930	.323	.072	.687	.144
Open	1152	21	32	26.23	1.599	.184	.072	− .043	.144
Agree	1152	21	32	26.28	1.575	.269	.072	− .214	.144
Consc	1152	15	29	20.98	3.334	− .194	.072	− .819	.144
Outcome	1152	71	86	79.37	1.779	.340	.072	1.252	.144
Valid N (listwise)	1152								

*Edulevel* Educational levels, *Extra* extraversion, *Neuro* neuroticism, *Open* openness to a new experience, *Agree* agreeableness, *Consc* conscientiousness, *Outcome* learning outcomes

### The linear regression analysis

We adopted linear regression analysis since linear regression could be used to establish the relationship between dependent and independent variables (Luo et al., 2020). The linear regression analysis (see Table 3) revealed that the demographic variables *gender* and *neuroticism* did not contribute significantly to the regression model for the dependent variable–learning outcomes [*t* (1,151) = 0.996, *p* = 0.319 for gender; *t* (1,151) = 1.696, *p* = 0.09 for neuroticism], while educational levels, extraversion, agreeableness, conscientiousness, and openness to a new experience contributed significantly to the regression model for the dependent variable–learning outcomes [*t* (1,151) = 2.548, *p* = 0.011 for educational levels; *t* (1,151) = − 3.817, *p* < 0.01 for extraversion; *t* (1,151) = 9.929, *p* < 0.01 for agreeableness; *t* (1,151) = 2.713, *p* = 0.007 for conscientiousness; *t* (1,151) = 6.993, *p* < 0.01 for openness to a new experience].

**Table 3:** Linear regression analysis results

Model	Unstandardized coefficients			Standardized coefficients	t	Sig
	B		Std. error	Beta		
(Constant)	57.604		.891		64.646	.000
Gender	.071		.071	.020	.996	.319
Edulevel	.193		.076	.054	2.548	.011
Extra	− .075		.020	− .076	− 3.817	.000
Neuro	.034		.020	.037	1.696	.090
Open	.340		.049	.305	6.993	.000
Agree	.499		.050	.442	9.929	.000
Consc	.030		.011	.057	2.713	.007
Regression		F		212.57	Sig	.00
R square		.565		Adjusted R square	.563	
Dependent variable: learning outcomes						

*Edulevel* Educational levels, *Extra* extraversion, *Neuro *neuroticism, *Open* openness to a new experience, *Agree* agreeableness, *Consc* conscientiousness, *Outcome* learning outcomes

Gender, educational levels, and general personality traits such as agreeableness, conscientiousness, neuroticism, and openness to a new experience explained 56.3% of the variance for learning outcomes, *F* (1,151) = 212.57, *p* < 0.01. Therefore, we rejected the first and fifth alternative hypotheses and accepted the other alternative hypotheses (see Table 4).

**Table 4:** Results of hypothesis testing

N	Hypothesis	Result
1	The gender of learners is significantly and strongly correlated with online learning outcomes	Rejected
2	The educational level is significantly and strongly correlated with online learning outcomes	Accepted
3	The personality is significantly and strongly correlated with online learning outcomes	Accepted
4	The level of extraversion is negatively correlated with online learning outcomes	Accepted
5	The level of neuroticism is negatively correlated with online learning outcomes	Rejected
6	The level of conscientiousness is positively correlated with online learning outcomes	Accepted
7	The level of agreeableness is positively correlated with online learning outcomes	Accepted
8	The level of openness to a new experience is positively correlated with online learning outcomes	Accepted

### Results from the interviews

The qualitative data obtained from the interviews produced results consistent with those from previous literature. The extraverts prefer the physical classrooms to the online learning contexts because the former may foster the interactions with peers and teachers, while the latter can inhibit their social interactions (Fuster, 2017). For example, an extroverted interviewee said that he would be delighted if the teacher’s skillful use of technologies could connect him directly to peers and teachers for interactions. Nevertheless, introverted interviewees voiced their preferences for the online pedagogy rather than the traditional face-to-face instruction.

The learners with a strong personality trait such as agreeableness tend to mutually trust and thus cooperate with peers and teachers. For example, an interviewee said, “I prefer to learn online to face-to-face methods because I can cooperate with my peers easily online.” This interviewee, measured via BFS, has strong agreeableness. Similarly, those with strong openness to a new experience tend to accept the technologies used in online learning, which is evidenced by an interviewee’s saying, “I like to learn new online technologies even it is difficult”. Measured by BFS, this interviewee has strong openness to a new experience. Most learners with strong conscientiousness also prefer online learning to traditional methods. Most of them thought that online learning could provide much more resources to them than the traditional method.

On the contrary, based on the interviewees’ opinions, extraverts prefer traditional face-to-face methods to online learning, leading to different learning outcomes. For example, an interviewee with strong extraversion tested by BFS said, “I hate learning online because I cannot make as many friends as in traditional learning contexts”. As expressed by an interviewee, learners with strong neuroticism do not like online learning. An interviewee with strong neuroticism said, “I feel nervous when learning online because I cannot really interact with my peers.”

The majority of females (> 80%) reported that they preferred a consistent learning method although they did not like the online learning approach, while most males (> 85%) preferred the online learning method to the traditional face-to-face method since the former was much more convenient than the latter. Neither males nor females reported any significant differences in online learning outcomes. A higher proportion of postgraduates than undergraduates reported their preferences for the online learning approach because they thought online learning could provide great freedom for their self-regulated learning.

## Discussion

### Educational levels

Undergraduates did not deem online learning as a most satisfactory instructional approach since they more positively evaluated teachers and course contents than the online videos. The reasons might be either that undergraduates were subject to the distractions of visual stimulation such as online videos or that they failed to spend enough time watching the online videos to acquire knowledge (Evans, 2014). They might have been surfing the Internet for entertainment or chatting with their friends. However, the postgraduates, with stronger self-regulation, might have been more resistant to the external disturbances and could keep their learning behaviors under control. They thus preferred the online learning method to the traditional method, resulting in higher learning outcomes than the undergraduates.

### Gender

Findings regarding gender differences in online learning outcomes tend to be inconsistent and even paradoxical. Online female learners prove more perseverant and engaged than males (Richardson & Woodley, 2003), while males tend to hold more stable positive attitudes toward online learning (Nistor, 2013). While females have stronger self-regulation than males in online learning contexts (Alghamdi et al., 2020), males can use more learning strategies and have better technical skills than females. The above findings may have offset the gender preferences in online learning, which might lead to no significant gender differences revealed in online learning outcomes. Rationales for inconsistent findings in gender differences may not be limited to the above. Future research could do more in-depth research into this field.

Teachers could design different courses for different genders. For females, teachers could design courses in need of more engagement and patience, while for males, teachers could provide courses in need of advanced technical skills and learning strategies. For those mixed with males and females, the teacher could strike a balance by providing various kinds of courses and tasks to attract their attention and improve their learning outcomes.

### Personality traits

The perception of learners’ different personalities could improve online learning effectiveness. Identification of personalities could allow teachers to better perceive students and to design more reasonable teaching strategies (Lai et al., 2020). Online learning could achieve success because students’ individual needs and preferences could be met by personalized methods in online contexts. Learners’ personalities require teachers to design adaptive teaching strategies and approaches to maximize students’ learning outcomes (Kratky et al., 2016).

In online learning contexts, it is hard for learners to acquire knowledge, enhance self-efficacy and use learning strategies without synchronous online teaching support since they need to decide what to learn, how to learn, and how much time is needed to learn (Mamun et al., 2020). If learners could not self-regulate their learning behaviors, the online system might be unable to facilitate their inquiry learning effectiveness (Jacobson, 2008).

Teachers could design different pedagogical approaches to cater for learners with different personalities. For those with strong neuroticism, teachers could design some interesting contents to release their negative emotions, reduce their stress, and relax them. For those with a strong extroversion trait, teachers could provide them with opportunities for interpersonal communication and design interactive academic activities for them. For those with strong personalities such as agreeableness, conscientiousness, and openness to a new experience, teachers could increase the amount of knowledge using updated technologies, raise the level of difficulty of knowledge, and establish a higher learning goal than those with traits of neuroticism and extroversion.

## Conclusion

This concluding section consists of major findings, limitations of this study, and future research directions.

### Major findings

This study revealed the effect of educational levels, gender, and personality traits on online learning outcomes, especially during the COVID-19 pandemic. This study could provide a meaningful reference for online teachers and instructors to improve the effectiveness of online instruction.

### Limitations

There are two limitations to this study. On one hand, the participants were limited to China rather than other areas in the world. On the other hand, this study was conducted during the COVID-19 pandemic, which might not be generalizable to other contexts.

### Future research directions

Future online learning design could improve interpersonal interactions and encourage learners to post words in the online discussion forum. Interpersonal interactions could strongly and positively influence learners’ grades, whereas their learning outcomes were significantly influenced by course organization and presentation, learning objectives and assessments, and technology (Jaggars & Xu, 2016). The most robust indicator of online learning success was the number of words learners typed in the discussion forum rather than their personalities (Abe, 2020).

Future research could focus on how to design scaffolding online learning and how to improve the quality and dynamic of the online contents. Scaffolding learning could weaken the need of online teacher support by encouraging online self-regulated learning (Mamun et al., 2020). The quality and dynamics were more important factors influencing the learning effect than mere online discussion (Davies & Graff, 2005).

Future research could also highlight blended learning rather than either merely online or traditional face-to-face learning. Forty-five studies have recently compared the effectiveness between online, blended, and traditional pedagogy in higher education, which revealed the online instruction was especially effective when combined with the blended instruction (Means et al., 2013a, 2013b). Pure online learning led to significantly lower grades (Hung et al., 2012) and passing rate (Freidhoff, 2017) than traditional face-to-face learning.

## Data Availability

We uploaded the data in the submission system.

## Appendix A. A Big Five Inventory (McCrae & Costa, 1995)

**Table:**

I see myself as someone who:	
*Factor I extraversion*	
1. Warms up quickly to others	1. Disagree strongly 2. Disagree a little 3. Neither agree nor disagree 4. Agree a little 5. Agree strongly
2. Prefers to be alone	
3. Is always on the go	
4. Can talk others into doing things	
5. Seeks quiet	
6. Is assertive and takes charge	
7. Holds back from expressing my opinions	
8. Enjoys being part of a group	
9. Lets things proceed at their own pace	
*Factor II neuroticism*	
10. Often feels blue	1. Disagree strongly 2. Disagree a little 3. Neither agree nor disagree 4. Agree a little 5. Agree strongly
11. Is not easily bothered by things	
12. Becomes stressed out easily	
13. Becomes overwhelmed by emotions	
14. Is calm, even in tense situations	
15. Is afraid that I will do the wrong thing	
16. Keeps my cool	
17. Does things I later regret	
*Factor III openness to a new experience*	
18. Does not have a good imagination	1. Disagree strongly 2. Disagree a little 3. Neither agree nor disagree 4. Agree a little 5. Agree strongly
19. Loves to read challenging material	
20. Is interested in many things	
21. Tries to understand myself	
22. Is not interested in abstract ideas	
23. Believes in the importance of art	
24. Prefers to stick with things that I know	
25. Tends to vote for conservative political candidates	
*Factor IV agreeableness*	
26. Suspects hidden motives in others	1. Disagree strongly 2. Disagree a little 3. Neither agree nor disagree 4. Agree a little 5. Agree strongly
27. Trusts others	
28. Contradicts others	
29. Values cooperation over competition	
30. Is easy to satisfy	
31. Thinks highly of myself	
32. Is concerned about others	
33. Puts people under pressure	
*Factor V conscientiousness*	
34. Completes tasks successfully	1. Disagree strongly 2. Disagree a little 3. Neither agree nor disagree 4. Agree a little 5. Agree strongly
35. Often makes last‐minute plans	
36. Excels in what I do	
37. Often forgets to put things back in their proper place	
38. Postpones decisions	
39. Works hard	
40. Pays my bills on time	
41. Doesn’t see the consequences of things	
